# A Commentary on “Art Therapy Interventions for Syrian Child and Adolescent Refugees: Enhancing Mental Well-being and Resilience”

**DOI:** 10.1007/s11920-025-01609-5

**Published:** 2025-04-29

**Authors:** Simona T. Milev

**Affiliations:** Syracuse University, Syracuse, NY USA

The paper “Art Therapy Interventions for Syrian Child and Adolescent Refugees: Enhancing Mental Well-being and Resilience” by Al-Hroub et al. (2023) illuminates the significance of creative art therapies for supporting the mental wellness of young refugees. Addressing essential themes such as overcoming trauma, developing resilience, and expressing cultural identity, this study highlights the ability for art to overcome linguistic and cultural barriers in a movement for positive change. We deeply appreciate the authors of this study for their valuable contributions to the field of art therapy and mental wellbeing (and further, psychiatric disorders). Yet, this paper also provides an opportunity to highlight just how much more we have to learn about the therapeutic role of art, and to reinforce the importance of addressing some lingering needs in the field.

First and most significantly, the authors decided on a relatively non-directive, open studio approach to art therapy by providing the participants different kinds of media with which they could choose to express themselves. The authors raise a meaningful point that the increased variety of options allowed the participants to feel more comfortable expressing themselves in whatever way they wished; however, the lack of a guided project and subsequent discussion potentially risks underutilizing all of the potential benefits of this therapy.

It is crucial for the field to continue exploring the distinction between directive and non-directive art therapy. While the former entails the art therapist providing patients with a structured guide for a project, the latter often leaves patients to their own devices to create whatever comes to mind (the method adopted in the study of Al-Hroub et al. 2023). A non-directive approach certainly has its benefits, promoting an individual’s autonomy to freely express themselves and more easily allowing the flow of unconscious thoughts. However, many participants in art therapy need more guidance in the creative process, and not all artworks will be successful in directly addressing specific therapeutic targets for improved mental health. More specifically, if an art therapist were aiming to help participants think about a specific dimension of their mental health, letting them draw or paint something random may not be most conducive to this goal. A directive approach might more effectively make up for these limitations through its added structure and targeted goals, which allows the art therapists to be more of a mediator in the art-to-wellbeing process.

While the authors’ choice for a non-directive approach clearly shows benefits for the participants, a broader consideration on the potential benefits of both types of art therapy opens the discussion to how directive therapy could also play a role in Al Hroub et al.’s study and others like it. It is also important to consider that the relative success of different art therapy modalities may vary depending on the type of mental health or psychiatric conditions and symptoms participants face. Although there is not much current research on art therapy specifically, the use of non-directive methods has previously been found to be more or less effective depending on the type of psychiatric disorder tested. For example, some data suggest that non-directive methods are more beneficial for patients with post-traumatic stress disorder (PTSD), while not being as significantly effective for those with bipolar disorder (Yao & Kabir, 2023). While some research has been done in this area, the general dearth of studies on the relationship between specific kinds of approaches in art therapy and psychiatric disorders also indicates that more work of this type is sorely needed. Art therapy is also used in non-clinical settings with non-clinical populations as a means of potentially enhancing well-being and reducing stress; however, the relative effectiveness of directive and non-directive approaches in these settings has not been evaluated empirically.

There is a notable lack of research on child populations, another laudable aspect of this work by Al Hroub et al. It is crucial that the link between art therapy and mental health is intricately explored in both adults *and* children due to the differences in their expression of symptoms and daily experiences with psychiatric disorders, as well as the potential for developmental discontinuity across the lifespan. Since the benefits of art therapy can be applied to both child and adult populations, we should examine the parameters of treatment at various developmental stages to learn how to best administer this kind of treatment moving forward.

And just as art therapy should be applied to both children and adults, we must also pay the same attention to the distinction between refugee and inpatient populations. While Al Hroub et al. focus mainly on refugees in their article, our comments on the application of art therapy include not only refugees but also beyond it to wherever art therapy can be applied—in this case, our main focus is on outreach for psychiatric patients due to the mental health benefits this therapy has been found to promote.

Particularly, the field of art therapy would greatly benefit from a head-to-head comparison between various kinds of psychiatric disorders and the efficacy of using directive, non-directive, or a combination of both kinds of art therapy. We would like to take this opportunity to advocate for a randomized controlled clinical trial where participants undergo long-term art therapy sessions that implement these various techniques, before and after which their overall mental wellbeing and specific psychiatric symptoms are assessed. A thorough study such as this will play a pivotal role in improving our understanding of how to best implement art therapy for many kinds of prominent psychiatric disorders. We extend once more a strong appreciation of the work of Al Hroub et al., and hope that the attention it receives can be leveraged to sharpen the field’s direction and develop even better methods and understanding of patients’ needs in the future.

## Data Availability

No datasets were generated or analysed during the current study.

## References

[CR1] Al-Hroub, A. (2023). Art Therapy Interventions for Syrian Child and Adolescent Refugees: Enhancing Mental Well-being and Resilience. Current Psychiatry Reports, 25(12), 857–863.10.1007/s11920-023-01474-0 10.1007/s11920-023-01474-0 37943430

[CR2] Yao, L., & Kabir, R. (2025). Person-Centered Therapy (Rogerian Therapy). In StatPearls. StatPearls Publishing. http://www.ncbi.nlm.nih.gov/books/NBK589708/36944012

